# Post-Operative Radiotherapy for Soft Tissue Sarcoma of the Anterior Compartment of the Thigh: Should the Sartorius Muscle be Included?

**DOI:** 10.1080/13577140400025961

**Published:** 2005

**Authors:** Gillian C. Barnett, Andrew C. F. Hoole, Nicola Twyman, Sarah J. Jefferies, Neil G. Burnet

**Affiliations:** 1 Oncology Centre, Box 193 Addenbrooke's Hospital Hills Road Cambridge CB2 2QQ UK; 2 Department of Medical Physics Addenbrooke's Hospital Hills Road Cambridge CB2 2QQ UK; 3 University of Cambridge Department of Oncology Oncology Centre Addenbrooke's Hospital Hills Road Cambridge CB2 2QQ UK

## Abstract

*Purpose:* The clinical target volume (CTV) of post-operative radiotherapy for soft tissue sarcoma of the limbs conventionally includes the whole of the transverse cross-section of the affected anatomical compartment. In the anterior thigh sartorius appears to lie within its own fascial compartment and can be safely excluded. We investigated the potential impact of omitting sartorius from the anterior muscle compartment on patients with soft tissue sarcoma of the thigh.

*Patients and methods:*We used the planning CT data from six patients who had previously received post-operative radiotherapy for soft tissue sarcoma of the thigh. The anterior compartments were outlined twice, initially including and then excluding the sartorius muscle. The volumes of the anterior compartment (i.e., the CTVs), both with and without sartorius, and the corresponding planning target volumes (PTVs) were calculated. Treatment plans were prepared for each PTV. For both volumes the unirradiated normal tissue corridor was outlined on each CT slice. The volume and circumference of the unirradiated corridor were then calculated.

*Results:* For all six patients there was an important improvement in normal tissue sparing by excluding sartorius. The mean reduction in volume of the anterior compartment when sartorius was excluded was 10% (95% Confidence Interval 8–12%), whilst the mean decrease in PTV was 11% (95% CI 7–14%). There was a substantial increase in the volume of the unirradiated normal tissue corridor, with a mean value of 77% (95% CI 41–114%) when sartorius was excluded. In addition, the percentage increase in the size of the unirradiated normal tissue corridor, expressed as a percentage of the whole leg circumference, was 10% (95% CI 8–13%). When sartorius was included in the anterior compartment, the circumference of the unirradiated corridor was less than one-third of the whole leg circumference in four of the six patients. When sartorius was excluded, the circumference of the unirradiated corridor was greater than one-third of the leg circumference over the entire length of the target volume in all patients.

*Discussion:* It is essential to know the anatomy of the sartorius muscle to be able to exclude it from the anterior compartment. The increase in the size of the normal tissue corridor when sartorius is excluded should deliver clinical advantage by decreasing the normal tissue adverse effects.


*Sarcoma*, March/June 2005; 9(1/2): 1–6

Taylor & Francis

Taylor & Francis Group

**ORIGINAL ARTICLE**

### Post-operative radiotherapy for soft tissue sarcoma of the anterior compartment of the thigh: should the sartorius muscle be included?

GILLIAN C. BARNETT[1], ANDREW C. F. HOOLE[2], NICOLA TWYMAN[2], SARAH J. JEFFERIES[1], & NEIL G. BURNET[1,3]

[1]*Oncology Centre, Addenbrooke's Hospital, Hills Road, Cambridge, CB2 2QQ, UK,* [2]*Department of Medical Physics, Addenbrooke's Hospital, Hills Road, Cambridge, CB2 2QQ, UK and* [3]*University of Cambridge Department of Oncology, Oncology Centre, Addenbrooke's Hospital, Hills Road, Cambridge, CB2 2QQ, UK*

**Abstract**
*Purpose:* The clinical target volume (CTV) of post-operative radiotherapy for soft tissue sarcoma of the limbs conventionally includes the whole of the transverse cross-section of the affected anatomical compartment. In the anterior thigh sartorius appears to lie within its own fascial compartment and can be safely excluded. We investigated the potential impact of omitting sartorius from the anterior muscle compartment on patients with soft tissue sarcoma of the thigh.
*Patients and methods:* We used the planning CT data from six patients who had previously received post-operative radiotherapy for soft tissue sarcoma of the thigh. The anterior compartments were outlined twice, initially including and then excluding the sartorius muscle. The volumes of the anterior compartment (i.e., the CTVs), both with and without sartorius, and the corresponding planning target volumes (PTVs) were calculated. Treatment plans were prepared for each PTV. For both volumes the unirradiated normal tissue corridor was outlined on each CT slice. The volume and circumference of the unirradiated corridor were then calculated.
*Results:* For all six patients there was an important improvement in normal tissue sparing by excluding sartorius. The mean reduction in volume of the anterior compartment when sartorius was excluded was 10% (95% Confidence Interval 8–12%), whilst the mean decrease in PTV was 11% (95% CI 7–14%). There was a substantial increase in the volume of the unirradiated normal tissue corridor, with a mean value of 77% (95% CI 41–114%) when sartorius was excluded. In addition, the percentage increase in the size of the unirradiated normal tissue corridor, expressed as a percentage of the whole leg circumference, was 10% (95% CI 8–13%). When sartorius was included in the anterior compartment, the circumference of the unirradiated corridor was less than one-third of the whole leg circumference in four of the six patients. When sartorius was excluded, the circumference of the unirradiated corridor was greater than one-third of the leg circumference over the entire length of the target volume in all patients.
*Discussion:* It is essential to know the anatomy of the sartorius muscle to be able to exclude it from the anterior compartment. The increase in the size of the normal tissue corridor when sartorius is excluded should deliver clinical advantage by decreasing the normal tissue adverse effects.

**Key words:** *Sarcoma, radiotherapy, post-operative*

### Introduction

Post-operative radiotherapy for soft tissue sarcoma of the limb conventionally involves irradiation of the entire transverse cross-section of the affected anatomical compartment. Inclusion of sartorius in the anterior compartment of the thigh will result in a larger clinical target volume for patients receiving radiotherapy after surgical removal of an anterior tumour. This is especially marked because the sartorius muscle becomes progressively more medial and posterior as it runs distally, and therefore determines the posterior limit of the anterior compartment in the lower half of the thigh. If sartorius is excluded from the anterior compartment, parallel opposed fields used to deliver the radiotherapy can be shifted anteriorly. This means that considerably less normal tissue is treated (Figure 1).

An anatomical investigation was performed in our institution to discover whether the sartorius muscle

Correspondence to: Gillian C. Barnett, Oncology Centre, Box 193, Addenbrooke's Hospital, Hills Road, Cambridge, CB2 2QQ, UK. E-mail: gillbarnett@doctors.org.uk

ISSN 1357–714X print/ISSN 1369–1643 online © 2005 Taylor & Francis Group Ltd
DOI: 10.1080/13577140400025961

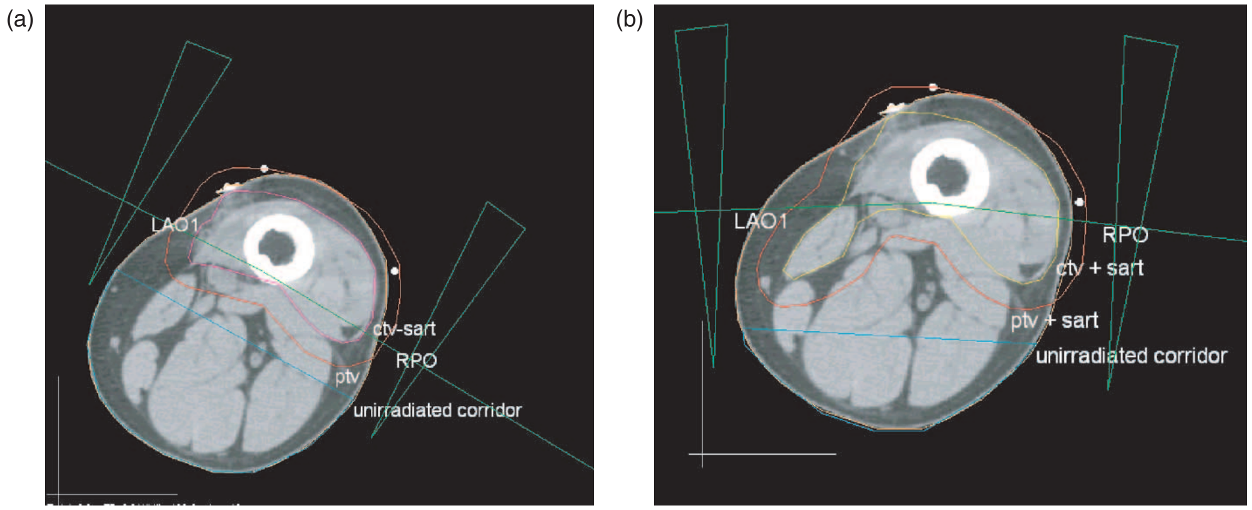

Figure 1. (a) Radiotherapy plan with sartorius excluded from the CTV. LAO1 and RPO represent the left anterior oblique and right posterior oblique field, respectively. (b) Radiotherapy plan with sartorius included in the CTV. LAO1 and RPO represent the left anterior oblique and right posterior oblique field, respectively.

is surrounded by a fascial membrane [1]. In all cases sartorius was enclosed within a fascial sheath of its own.

As a result of this anatomical investigation, we sought to evaluate the effect of omitting sartorius from the CTV for anterior compartment radiotherapy. The CT data of six patients with sarcoma were reviewed and radiotherapy plans were prepared to treat the anterior compartment of the thigh including and excluding sartorius.

### Patients and methods

The radiotherapy planning CT scans of six patients with soft tissue sarcoma of the limb were reviewed. Four of these patients had in fact been treated for posterior compartment tumours. Five of the six patients were male. The mean age of the patients was 59.8 years (range 40–78 years).

For each patient, we outlined the anterior compartment on each of the CT slices, initially including and then excluding the sartorius muscle. This anterior compartment was therefore assumed to be the clinical target volume (CTV). The length of the CTV was assumed to be the same as that of the original plan. The planning target volume was then created by expanding the CTV by 1 cm isotropically. Full conformal radiotherapy plans were prepared for each patient, including and excluding the sartorius muscle. The planning system used was our in-house Addenbrooke's Radiotherapy Planning System (ARPS). An additional tool was written into ARPS to enable measurement of the circumference of the thigh and normal tissue corridor.

The volume of the anterior compartments and corresponding PTVs, including and excluding sartorius, were calculated for each patient. The unirradiated normal tissue corridor was outlined on each CT slice and defined as the volume of normal tissue, outside the projections of the treatment beams or conformal blocks, which should receive less than 50% of the prescribed dose. The corridor was then evaluated in two ways. Firstly, the total volume of this corridor was calculated. Secondly, the length of the skin surface in the unirradiated corridor at each level was measured and expressed as a percentage of the total leg circumference at that level. Figure 1 demonstrates the radiotherapy plans with sartorius excluded and included in the CTV.

Having measured the volumes of the anterior compartment, PTV and unirradiated corridor with and without sartorius, the change in volume for each patient as a result of omitting sartorius could be calculated. The mean increase in the skin length of the unirradiated corridor, expressed as a percentage of the whole leg circumference, was also calculated.

The mean changes in volume and circumference for the six-patient group were calculated. Confidence intervals were calculated using the binomial approximation.

### Results

Figure 2 shows the volumes of the anterior compartment and planning target volume, both including and excluding sartorius, for the six patients. The volumes of the unirradiated corridor when sartorius is included and excluded are displayed in Table I. The changes in the length of the skin surface of the unirradiated corridor, expressed as a percentage of the whole leg circumference, are displayed in Table II. It can be seen that, in four out of six cases, standard planning would have spared less than one-third of the circumference of the leg. Exclusion of sartorius

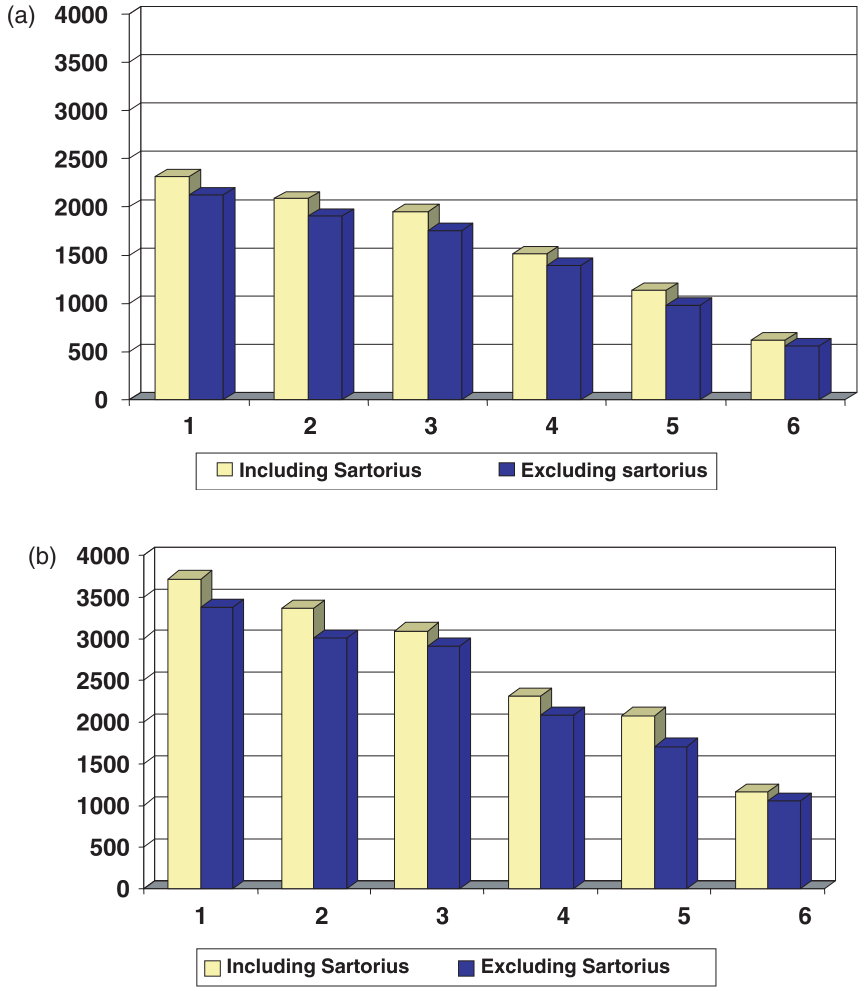

Figure 2. (a) The volume of the anterior compartment when sartorius is included and excluded from the anterior compartment. (b) The volume of the planning target volume when sartorius is included and excluded from the anterior compartment.

Table I. The changes in volume of the normal tissue corridor when sartorius is included and excluded from the anterior compartment.

| | Volume of unirradiated corridor (ml) | | |
|---|---|---|---|
| Patient | With sartorius | Without sartorius | Percent increase[1] |
| 1 | 523 | 1226 | 134 |
| 2 | 1584 | 2238 | 41 |
| 3 | 2190 | 3161 | 44 |
| 4 | 1644 | 2888 | 76 |
| 5 | 571 | 1328 | 133 |
| 6 | 751 | 1024 | 36 |

[1]Percentage increase in the volume of the unirradiated corridor when sartorius is excluded from the anterior compartment.

increased the proportion of the circumference spared to greater than one-third in all cases.

The mean changes in volume and circumference are summarised in Table III. It can be seen that the exclusion of sartorius from the anterior compartment results in a 10% (95% CI 8–12%) decrease in the volume of this compartment. This corresponds to a large increase in volume of the unirradiated corridor; the mean for the six patients was 77% (95% CI 41–114%). The increase in the means of the normal tissue corridor, expressed as a percentage of the whole leg circumference, was 10% (95% CI 8–13%). The range of these means was 5–15%.

### Discussion

The results of this study add to the findings of the anatomical investigation undertaken in our department [1]. This investigation, though limited to eight legs in six individuals, demonstrated that the intermuscular septum does lie posterior to sartorius and that sartorius is enclosed within a fascial sheath of its own. It also confirmed the very thick nature of the femoral sheath fascia, which would protect sartorius above the adductor canal from invasion by a tumour sited deep and posterior to it.

In all eight legs in the six individuals, there was evidence of a fascial envelope around sartorius in the upper thigh. Posteriorly, this is reinforced by the thick fascia of the femoral sheath. In the lower half of the thigh, in four out of six individuals, there was clear evidence that this envelope continued distally.

Table II. The changes in skin circumference of the normal tissue corridor when sartorius is included and excluded from the anterior compartment.

| | Skin circumference of unirradiated corridor (expressed as percentage of whole leg circumference) | | | | | | | | |
|---|---|---|---|---|---|---|---|---|---|
| | With sartorius | | | Without sartorius | | | Percent increase[1] | | |
| Patient | Min | Mean | Max | Min | Mean | Max | Min | Mean | Max |
| 1 | 6 | 25 | 44 | 27 | 36 | 51 | 5 | 11 | 21 |
| 2 | 0 | 27 | 44 | 27 | 37 | 44 | 0 | 10 | 28 |
| 3 | 23 | 35 | 44 | 36 | 44 | 51 | 6 | 9 | 14 |
| 4 | 15 | 31 | 41 | 38 | 42 | 49 | 4 | 11 | 22 |
| 5 | 9 | 26 | 36 | 35 | 41 | 48 | 7 | 15 | 26 |
| 6 | 34 | 40 | 45 | 42 | 45 | 49 | 0 | 5 | 9 |

[1]Percentage increase in the skin circumference of the unirradiated corridor when sartorius is excluded from the anterior compartment.

Table III. Mean changes for the six-patient group in volume and circumference when sartorius is excluded from the anterior compartment.

| | Sartorius excluded | 95% Confidence intervals |
|---|---|---|
| Mean reduction in volume of anterior compartment | 10% | 8–12% |
| Mean reduction in PTV | 11% | 7–14% |
| Mean increase in volume of unirradiated corridor | 77% | 41–114% |
| Mean increase in circumference of unirradiated corridor | 10% | 8–13% |

In the lower half of the thigh in two subjects the femoral sheath fascia appeared to merge with an aponeurotic layer on the surface of vastus medialis. It is less definite that this layer would prevent invasion of tumour, although qualitatively this layer appeared to be as thick as the intermuscular septum, suggesting that it could resist tumour invasion as well as the septum.

Both studies suggest that the exclusion of the sartorius muscle from the anterior compartment results in a significant reduction in normal tissue irradiated. This can be shown as an increase in both the volume and circumference of the unirradiated normal tissue corridor. This should therefore lead to decreased normal tissue complications.

Several studies have shown that the functional outcome after radiotherapy to the limb is related to the radiotherapy volume and the size of the unirradiated corridor [2–5]. The total dose and dose per fraction of radiotherapy used is also a significant factor in determining range of movement, muscle power and limb function [2,4,6]. Stinson *et al.* [2] reviewed 145 patients who underwent combined modality limb sparing therapy at the National Cancer Institute for extremity soft tissue sarcoma between 1975 and 1986. Oedema and tissue induration was more common with increasing field length. Chronic ulcer or infection was more frequently seen in patients with lower extremity tumours when more than 75% of the extremity diameter was irradiated. Karasek *et al.* [3] found that the volume irradiated to greater than 55 Gy significantly correlated with post-treatment morbidity.

It has been recommended that the normal tissue corridor should comprise at least one-third of the circumference of the limb [7]. This was achieved over the entire length of the target volume in all six patients after the sartorius muscle was excluded from the anterior compartment.

The importance of post-treatment function is paramount in the management of soft tissue sarcoma. A study of limb function and quality of life in 40 patients with soft tissue sarcoma of the head and neck, trunk, upper and lower extremity showed that patients with lower limb tumours had the greatest functional impairment [8]. A randomised trial comparing preoperative and postoperative radiotherapy used three conceptually different outcome measures to evaluate function and health status [9]. The presence of a lower extremity tumour was predictive for a greater change in both the Musculoskeletal Tumour Rating Scale (MSTS) and the Toronto Extremity Salvage Score (TESS). These outcome measures are evaluated by the clinician and patient, respectively. This study also suggests that both dose and field size are important factors in determining morbidity and function.

Although the functional consequences of limb conserving treatment are good, the addition of radiotherapy or chemotherapy to surgery has an adverse impact. This may be especially significant if both radiotherapy and chemotherapy are required. It is therefore essential that the target volume and dose of radiotherapy be optimised. Quality of life and limb function depend on achieving good local control, radiotherapy dose and technique [10,11].

In recent years some centres have changed from the conventional practice of irradiating the entire anatomical department to the use of planning margins of 4–5 cm around the GTV in the transverse plane. This is partly because high local control rates have been achieved with brachytherapy, despite tighter margins around the tumour [12–14]. Alektiar *et al.* [12] determined the target region by adding 2.0 cm to the superior and inferior dimensions of the tumour bed, with 1.5–2.0 cm added in the medial and lateral directions, achieving local control rates of 91% (95% CI 86–96%) with lower extremity high grade lesions. Karakousis *et al.* [15] have shown local control rates of 81% for selected high grade adult soft tissue sarcomas, resected with a relatively tight 2-cm clear margin, without the addition of adjuvant radiotherapy. The same group has reported a local control rate of 76% in those considered to require radiotherapy after resection. In contrast, Mundt *et al.* [6] report a local rate of 93% with margins of 5 cm or more, compared to 30% with margins less than 5 cm. Other experts also favour wider margins [7,16], so this issue remains unresolved.

Our study is small and the confidence intervals, particularly for the change in volume of the unirradiated corridor, are large. However, it can be seen that in all cases there was a considerable increase in the volume of the unirradiated corridor when sartorius was excluded. Because four of the patients had previously been treated for posterior compartment tumours, it is possible that the amount of normal tissue sparing has in fact been underestimated in this study. By excluding the sartorius muscle, we can therefore decrease the size of the anterior compartment. Since we have shown that sartorius is protected by its own fascial sheath, the muscle does not need to be included in the treatment field for a low posterior tumour.

In our centre, sartorius is no longer included routinely in the CTV for anterior compartment soft tissue sarcomas. However, in some clinical situations it may be necessary to include the muscle. For example, sartorius must be included in the CTV if pre-operative imaging suggests involvement of this muscle. If there is any doubt about whether sartorius is involved, it would appear prudent to include the muscle in the CTV. In a seventh patient, who was not included in the study, it was not possible to define the sartorius muscle as it appeared to merge with gracilis at the junction of the upper and middle third of the thigh. In this patient the tumour was located medially and therefore the medial scar may have altered the tissue plane. Anatomy definition is improved on MRI scanning and this imaging modality can be very helpful when available. MRI/CT fusion on modern planning systems may help to accurately contour patients where the CT images are not clear.

### Acknowledgments

We are grateful to Mr R. J. Bridger and Dr R. H. Harmer for logistical support.

### References

[1] Burnet NG, Bennet-Britton T, Hoole ACF, Jefferies SJ, Parkin IG. The anatomy of sartorius muscle and its implications for sarcoma radiotherapy. Sarcoma 2004;8(1):7–12.

[2] Stinson SF, DeLaney TF, Greenberg J, Yang JC, Lampert MH, Hicks JE, Venzon D, White DE, Rosenberg SA, Glatstein EJ. Acute and long-term effects on limb function of combined modality limb sparing therapy for extremity soft tissue sarcoma. Int J Radiat Oncol Biol Phys 1991;21(6):1493–1499.

[3] Karasek K, Constine LS, Rosier R. Sarcoma therapy: functional outcome and relationship to treatment parameters. Int J Radiat Oncol Biol Phys 1992;24(4):651–656.

[4] Robinson MH, Spruce L, Eeles R, Fryatt I, Harmer CL, Thomas J, Westbury G. Limb function following conservative treatment of adult soft tissue sarcoma. Eur J Cancer 1991;27(12):1567–1574.

[5] Robinson MH, Bidmead AM, Harmer CL. Value of conformal planning in the radiotherapy of soft tissue sarcoma. Clin Oncol 1992;4(5):290–293.

[6] Mundt AJ, Awan A, Sibley GS, Simon M, Rubin SJ, Samuels B, Wong W, Beckett M, Vijayakumar S, Weichselbaum RR. Conservative surgery and adjuvant radiation therapy in the management of adult soft tissue sarcoma of the extremities: clinical and radiobiological results. Int J Radiat Oncol Biol Phys 1995;32(4):977–985.

[7] Swedish Council on Technology Assessment in Health Care (SBU). Radiotherapy for Cancer. Acta Oncol 1996;35 (Suppls 6:1–100 and 7:1–152).

[8] Lampert MH, Gerber LH, Glatstein E, Rosenberg SA, Danoff JV. Soft tissue sarcoma: functional outcome after wide local excision and radiation therapy. Arch Phys Med Rehabil 1984;65:477–480.

[9] Davis AM, O'Sullivan B, Bell RS, Turcotte R, Catton CN, Wunder JS, Chabot P, Hammond A, Benk V, Isler M, Freeman C, Goddard K, Bezjak A, Kandel RA, Sadura A, Day A, James K, Tu D, Pater J, Zee B. Function and health status outcomes in a randomised trial comparing preoperative and postoperative radiotherapy in extremity soft tissue sarcoma. J Clin Oncol 2002;20(22):4472–4477.

[10] Nielsen OS, Cummings B, O'Sullivan B, Catton C, Bell RS, Fornasier VL. Preoperative and postoperative irradiation of soft tissue sarcomas: effect of radiation field size. Int J Radiat Oncol Biol Phys 1991;Nov 21:1595–1599.

[11] Bell BG, Bell RS, Davis A, O'Sullivan B, Mahoney J, Manktelo RT, Bowen V, Catton C, Fornasier VL, Langer F. Wound healing complications after soft-tissue sarcoma surgery. Plast Reconstr Surg 1994;93:980–987.

[12] Alektiar KM, Leung D, Zelefsky MJ, Healey JH, Brennan MF. Adjuvant brachytherapy for primary high-grade soft tissue sarcoma of the extremity. Ann Surg Oncol 2002 Jan–Feb;9(1):48–56.

[13] Pister PW, Harrison LB, Leung DH, Woodruff JM, Casper ES, Brennan MF. Long-term results of a prospective ramdomized trial of adjuvant brachytherapy in soft tissue sarcoma. J Clin Oncol 1996;14(3):859–868.
[14] Habrand JL, Gerbaulet A, Pejovic MH, Contesso G, Durand S, Haie C, Genin J, Schwabb G, Flamant F, Albano M. Twenty years experience of interstitial brachytherapy in the management of soft tissue sarcomas. Int J Radiat Oncol Biol Phys 1991 Mar;20(3): 405–411.
[15] Karakousis CP, Zografos GC. Radiation therapy for high grade soft tissue sarcomas of the extremities treated with limb-preserving sugery. Eur J Surg Oncol 2002 Jun;28(4):431–436.
[16] Lartigau E, Kantor G, Taieb S, Vilain MO, Ceugnart L, Lagarde P, Penel N, Depadt G. Définition des volumes-cibles [GTV & CTV] dans les sarcomes des tissus mous des extrémités [Definition of target volumes in soft tissue sarcomas of the extremities]. Cancer Radiother 2001 Oct;5(5): 695–703.

